# Thoracoscopic surgery using omental flap for bronchopleural fistula

**DOI:** 10.1186/s40792-019-0563-3

**Published:** 2019-01-14

**Authors:** Hideki Endoh, Ryohei Yamamoto, Nobuhiro Nishizawa, Yukitoshi Satoh

**Affiliations:** 1 Department of Thoracic Surgery, Saku Central Hospital Advanced Care Center, 3400-28 Nakagomi, Saku, Nagano 385-0051 Japan; 2 Department of Thoracic Surgery, Kitasato University School of Medicine, 1-15-1 Kitasato Minami-ku,, Sagamihara, Kanagawa 252-0374 Japan

**Keywords:** Bronchopleural fistula, VATS, Omental flap

## Abstract

**Background:**

A bronchopleural fistula (BPF) can lead to empyema and death after pulmonary resection. A minor leakage from a BPF has been reported to be successfully closed endobronchially, although thoracoplasty is usually needed.

**Case presentation:**

A case of successful thoracoscopic BPF closure using an omental flap in a 74-year-old man with emphysema who developed a BPF after right lower lobectomy for lung cancer is reported. Reoperation was performed to close the BPF using an omental flap. After successful closure of the BPF, the empyema resolved with intravenous antibiotics.

**Conclusions:**

Thoracoscopic single-stage omentoplasty without thoracotomy might be a useful treatment method when a BPF is diagnosed early.

## Background

A bronchopleural fistula (BPF) is one of the most difficult complications after major lung surgery because it can decrease patients’ activities and quality of life and predispose them to severe infections, such as pyothorax and pneumonia, which have a high mortality. In cases that develop pyothorax/intrathoracic infections, fenestrations in the chest wall are usually created by resection of several ribs to allow drainage, and plastic surgery is required more than 6 months after the first re-operation.

A case of successful management of a BPF by a single-stage closure with an omental flap through video-assisted thoracic surgery (VATS), which enabled control of a *Pseudomonas aeruginosa* infection with antibiotic treatment for 2 weeks, is reported.

## Case presentation

A 74-year-old man was admitted for right lower lobectomy with lower mediastinal and hilar lymph node dissection for squamous cell carcinoma. He had pulmonary emphysema secondary to smoking more than 50 pack-years. He had no diabetes mellitus, no history of steroid intake, and had not received chemotherapy or radiotherapy.

**Fig. 1:**
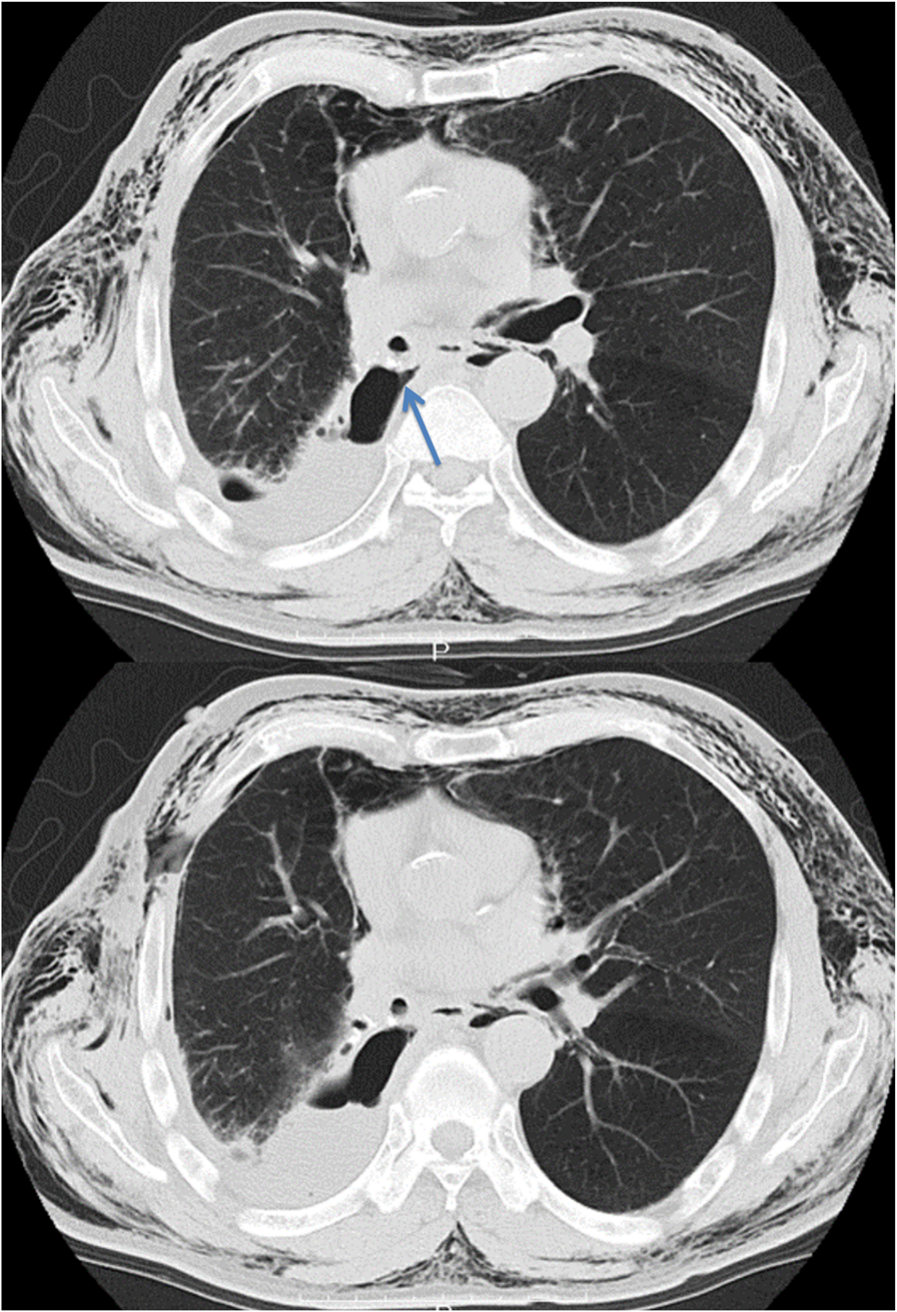
CT image before re-operation, on POD 12 following the primary operation (i.e., right lower lobectomy). The arrow shows the broken appearance of the bronchial stump. CT computed tomography, POD postoperative day

On postoperative day (POD) 10, the patient had pyrexia (38.4 °C), and C-reactive protein (CRP) was increased to 16.22 mg/dL. On POD 12, he developed subcutaneous emphysema. A BPF was suspected because of increasing air leakage through the chest tube and the broken appearance of the bronchial stump on chest computed tomography (Fig. 1).

### Operation

**Fig. 2:**
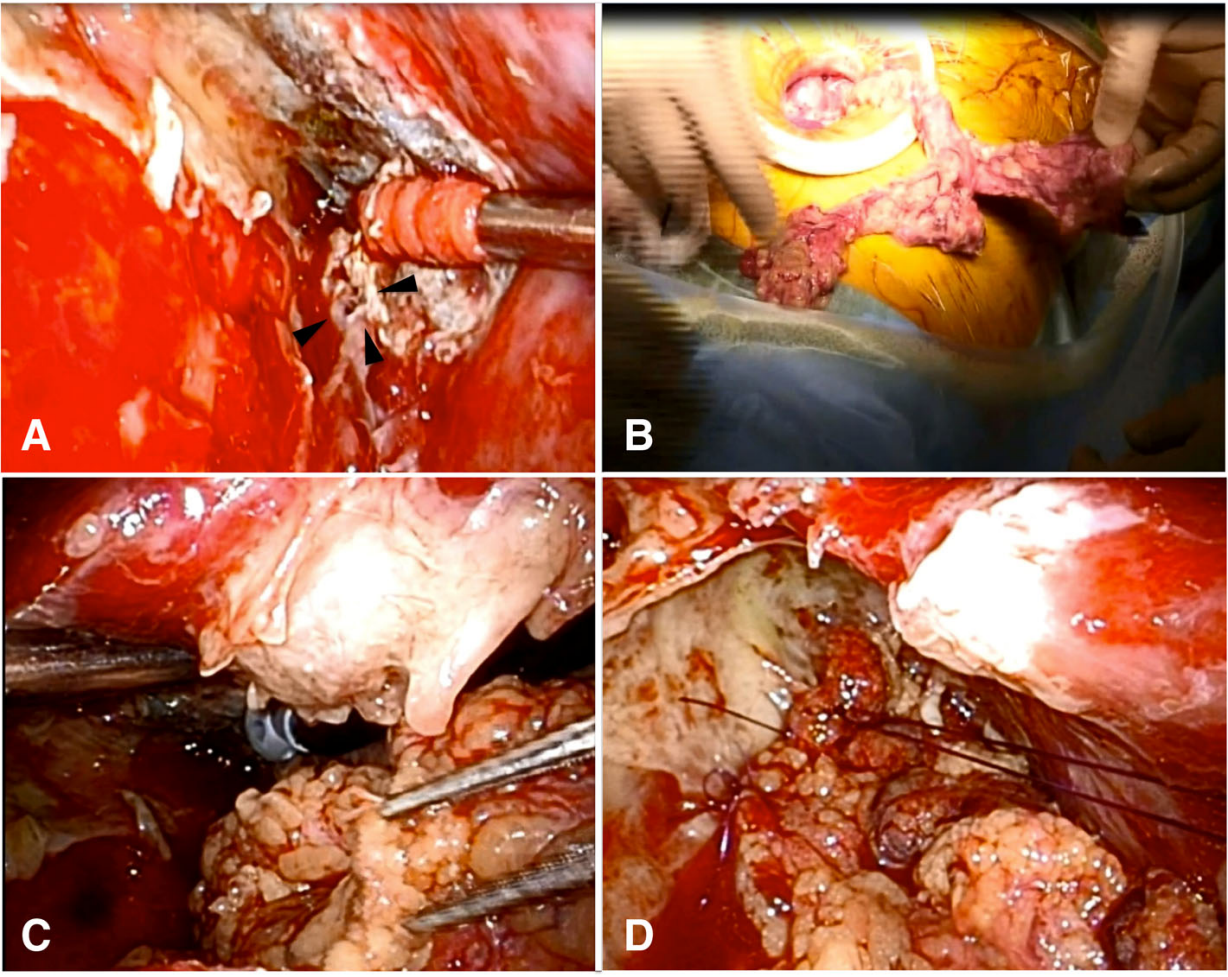
Surgical findings on re-operation. **a** The arrows indicate the bronchial fistula. **b** The omental flap during laparotomy. **c, d** The omental flap has been harvested and fixed in the thoracic cavity

On POD 13, reoperation was performed under general anesthesia. First, thoracoscopy in the lateral decubitus position confirmed the presence of the BPF, which was about 6–7 mm in diameter; the adhesions could be removed easily. Next, with the patient in the supine position, laparotomy was performed through a 7-cm skin incision; the right side of the omentum with a preserved right gastroepiploic artery was detached from the stomach for the omental flap. Lastly, with the patient back in the lateral position, the omental flap was led through the anterior mediastinum below the sternum and sutured above and below the bronchial fistula using two nonabsorbable mattress sutures. It was then fixed using three sutures to the parietal pleura without using fibrin sealant. A water test was not done, because the middle lobe held to the omental flap naturally and was expected to adhere soon. The fistula was covered with omentum and was not sutured directly for closure. Because the thoracic cavity had been narrowed due to inflammatory adhesions, and the working space was limited, the suturing technique was not straightforward. Finally, the BPF was covered with an omental flap. All procedures were done by VATS (Fig. 2). The thoracic space was washed using 2 L of saline, and the wound was closed with placement of an indwelling chest tube. The total surgical time was 4 h and 41 min, and blood loss was 100 mL.

**Fig. 3:**
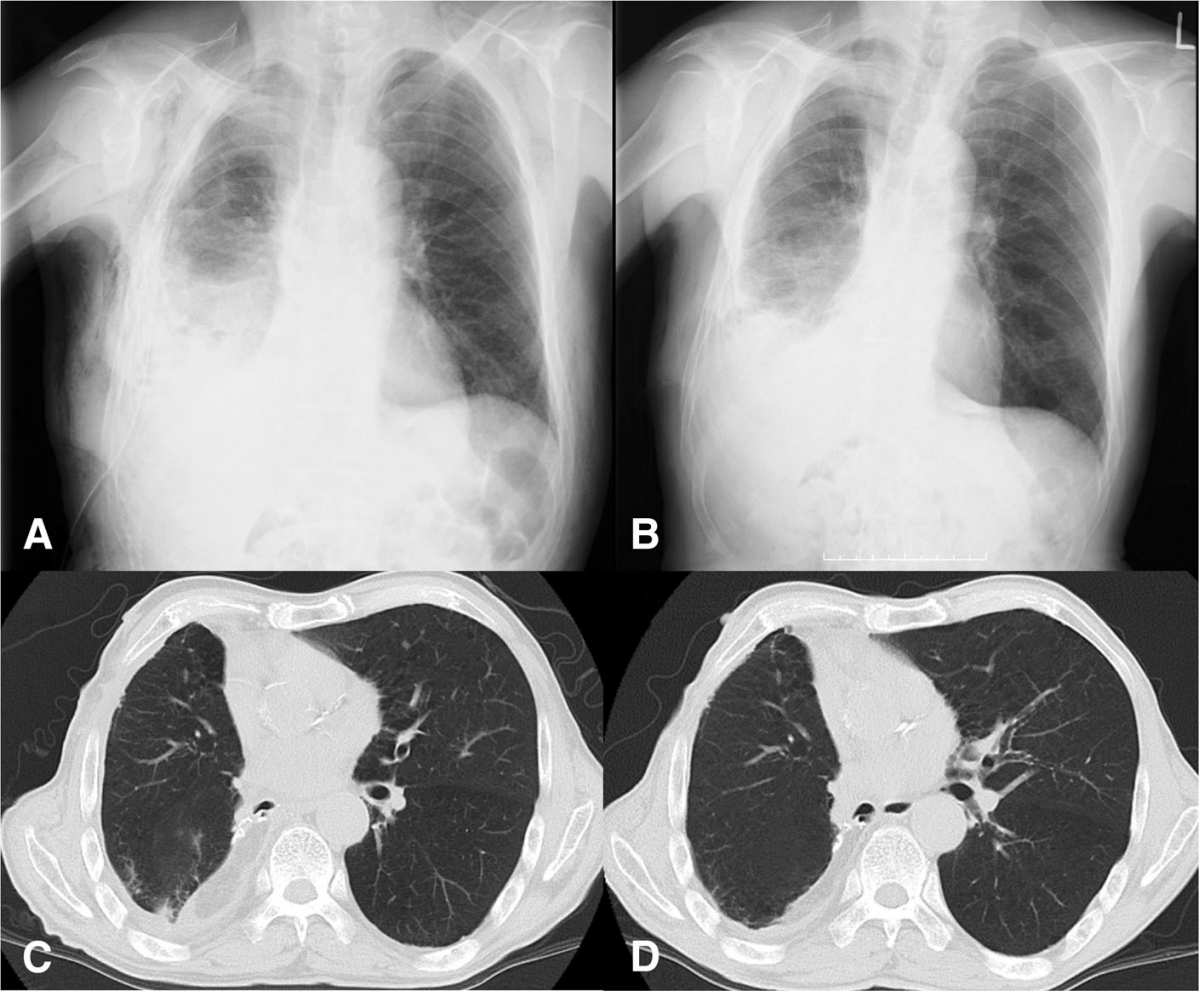
Findings after reoperation. After re-operation, the chest X-ray images on POD 2 (**a**) and POD 14 (**b**), as well as the chest CT images at 1 month (**c**) and at 3 months (**d**), are shown. CT computed tomography, POD postoperative day

After the reoperation, no air leakage was observed, and the chest drainage tube was removed on POD 4. The clinical decision was made based on the small quantity of drainage and the patient’s afebrile status, while still considering re-drainage if needed. Ceftazidime was administered intravenously for 2 weeks to treat the *P. aeruginosa* infection that was detected by cultures of the pleural effusion. The increased CRP level of 16.22 mg/dL before reoperation decreased to 7.19 mg/dL on POD 7 and to 1.64 mg/dL on POD 14. Although the patient complained of anorexia and pain for several days after the reoperation, his general condition was relatively better, and he was discharged 19 days after the reoperation (Fig. 3). At 2 years, he remained free from recurrence of cancer and infection.

## Discussion

The incidence of a BPF after lobectomy or pneumonectomy has been reported to be 1 to 4% [1, 2], and it carries a high risk of mortality or prolonged hospital stay. Uramoto and Hanagiri reported that primary closure of a BPF was successful in only 15.8% of cases, and the mortality rate was 57.9% [2]. Schneiter et al. reported the treatment outcomes of 75 patients with postpneumonectomy empyema, including 44 patients with a BPF [3]. The success rate after the first treatment was 86.7%, although the median number of interventions until final closure was 3.

Early diagnosis and early repair of BPFs are important. Although treatment options, such as endoscopic closure, have been reported [4], the usual choice of operation has been fenestration with open thoracotomy and partial resection of two to three ribs in order to control the empyema [5, 6]. In such cases, the gauze dressing needs to be changed after fenestration. The operator then needs to select between either suturing and closing the BPF or re-stapling the central bronchus of the fistula, resulting in pneumonectomy or bi-lobectomy; both methods need a muscle flap for closure. Park et al. reported the use of a serratus anterior musculocutaneous flap for BPF closure, with a mean operation time of 5 h and 32 min [6].

The omentum, rather than the intercostal muscle or latissimus dorsi muscle, has been considered to be better for flaps, not only because it promotes angiogenesis and healing, but also because of its anti-inflammatory role. Some surgeons recommend that omentoplasty be done as the first choice for empyema with/without a BPF [3, 7]. It should be noted that some patients complain of abdominal complications, such as diarrhea, anorexia, distention, and ileus, after omentoplasty [7]. Because the remodeling stage of healing begins 2–3 weeks after the onset of the lesion [8], an adequate drainage period after a second operation should be considered.

Nakajima et al. reported that single-stage closure may be appropriate when using a musculocutaneous flap [9]. They reported that single-stage closure without open treatment could be used in cases of good infection control by antibiotic administration and tube drainage. We believe that single-stage closure is appropriate for localized and early infections.

## Conclusions

Single-stage thoracoscopic omentoplasty without open thoracotomy (VATS omentoplasty) might be suitable for modern salvage surgery when treating acute empyema with a BPF after VATS lobectomy. The outcomes have fortunately been good, and although postsurgical drainage must be done more cautiously, this technique is appropriate in cases of BPFs that are diagnosed early and in those with a limited space affected by empyema.

## Abbreviations

BPF: Bronchopleural fistula
CRP: C-reactive protein
POD: Postoperative day
VATS: Video-assisted thoracic surgery
